# “Want to Learn” and “Can Learn”: Influence of Academic Passion on College Students' Academic Engagement

**DOI:** 10.3389/fpsyg.2021.697822

**Published:** 2021-06-17

**Authors:** Hui Zhao, Xiaoxian Liu, Chunhui Qi

**Affiliations:** Faculty of Education, Henan Normal University, Xinxiang, China

**Keywords:** academic engagement, academic passion, academic self-efficacy, teacher developmental feedback, college students

## Abstract

Although research exists on the relationship between passion and engagement among employees, the mechanisms of academic passion on academic engagement among students needs to be elucidated. Guided by the broaden-and-build and situated cognition theories, we explored the positive effect of academic passion on academic engagement, the mediating effect of academic self-efficacy, and the role of teacher developmental feedback as a moderator in the relationship between academic passion and academic engagement. Based on a sample of 1,029 college students from universities in the Henan Province of China, the results showed that academic passion was positively related to academic engagement, academic self-efficacy partially mediated the relationship between academic passion and academic engagement, and teacher developmental feedback effectively moderated the relationship between academic passion and academic engagement. These findings explained the mechanism underlying the relationship between academic passion and academic engagement. Moreover, the findings highlighted important factors that promote college students' academic engagement.

## Introduction

Academic engagement, defined as the degree of students' involvement in their studies, is indicated by the amount of energy they devote to studies (Stoeber et al., 2011). Consequently, Chinese educators now pay more attention to academic engagement in educational practice and evaluation because they play a key role in predicting positive academic performance and adaptive behaviors (Chen et al., 2015). It has been well-established that students with higher academic engagement tend to have higher academic achievement (Salanova et al., 2010; Carter et al., 2012) as well as lower dropout rates or lower incidences of misbehavior (Wang and Fredricks, 2014; Saeki and Quirk, 2015). Considering the importance of college students' academic engagement, this study intends to investigate the factors that affect academic engagement.

Anecdotal evidence of education indicates that passionate students are engaged students. As a psychological construct, passion is defined as a strong and explosive emotional state. Bonneville-Roussy et al. (2013) found that passion toward academics was positively associated with practice and the level of absorption displayed in academics. Based on the broaden-and-build theory, Fredrickson and Branigan (2001) proposed that learning-related positive emotions could expand the scope of one's cognition and activities as well as help one achieves one's goals. Researchers have investigated the relationship between job passion and work engagement among employees (Ho et al., 2011; Trépanier et al., 2014; Ho and Astakhova, 2018). However, few studies have examined the role of academic passion in the process of academic engagement (Stoeber et al., 2011). Moreover, the internal mechanism underlying this relationship remains unclear. Therefore, the role academic passion plays and how it affects academic engagement needs to be further explored.

According to the broaden-and-build theory, positive emotions can build resources such as long-term cognition (Fredrickson and Branigan, 2005). The question is, does academic passion affect academic engagement through cognitive processes? Academic self-efficacy is the basis for students to judge their abilities positively and meet the requirements of the academic environment (Oriol-Granado et al., 2017). Previous research has found that passion and academic self-efficacy are positively correlated (Drnovšek et al., 2014). In other words, self-efficacy can predict higher levels of academic engagement and improve academic performance (Oriol-Granado et al., 2017). However, whether academic self-efficacy plays a crucial cognitive role in the relationship between academic passion and academic engagement remains to be revealed. In situated cognition theory, knowledge is a situation-based activity, and cognition is a state constructed by the interaction between individuals and the environment (Brown et al., 1988). Teacher feedback is an important contextual factor, that helps students learn more effectively (Wang and Zhang, 2020), and research has shown that feedback can improve learners' engagement (Lewis et al., 2019). Therefore, to explore the mechanism by which academic passion affects academic engagement, we conducted a model with teacher developmental feedback as the moderator.

### Academic Passion and Academic Engagement

Academic engagement is a positive and fulfilling work-related state of mind that is characterized by vigor, dedication, and absorption in academics (Schaufeli et al., 2002). Furthermore, it is seen as the outcome of a process in which the school provides a social context that makes students feel competent, autonomous, and related (Skinner et al., 2008). While engagement may resemble passion, defined as a strong inclination toward an activity that individuals like (or even love) that they find important (Vallerand et al., 2007), it represents distinct constructs (Ho and Astakhova, 2018). The broaden-and-build theory explains that vigorous emotional experience is beneficial for expanding the scope of individual cognition and activities (Fredrickson and Branigan, 2005). In addition, according to the identity perspective in role investment theory, individuals devote cognitive attention, and time to the roles they are passionate about (Rothbard and Edwards, 2003). Several viewpoints of role investment theory create an expectation that people who are passionate about learning will display greater engagement in their studies. Empirical studies have found that because of increased demands-abilities fit and person-organization fit, passionate employees reported greater levels of job, and organizational engagement (Ho et al., 2011; Ho and Astakhova, 2018). Furthermore, harmonious passion is positively related to flow experience (Lavigne et al., 2012) and schoolwork engagement (Enwereuzor et al., 2016). In the academic field, passion has shown unique relationships with the central aspects of academic engagement (Stoeber et al., 2011; Lin et al., 2019). Therefore, based on previous evidence, we propose the following hypothesis:

Hypothesis 1: Academic passion is positively correlated with academic engagement.

### Mediating Role of Academic Self-Efficacy

According to Bandura (1997), self-efficacy is a judgment of self-competence that people use to perform tasks successfully. Academic self-efficacy is the extension and concretization of self-efficacy theory in the field of learning and is defined as students' judgments about whether they are capable of successfully reaching designated academic goals (Linnenbrink and Pintrich, 2003). Based on the broaden-and-build theory, passion may affect students' cognition of learning activities. Many studies have confirmed a positive and strong correlation between passion and self-efficacy (Baum and Locke, 2004; Drnovšek et al., 2014). Results have shown that positive emotions have a clear impact on academic performance, academic self-efficacy, and academic engagement (Oriol-Granado et al., 2017). This suggests that passion plays a key role in obtaining students' high self-efficacy. In addition, studies have revealed that self-efficacy is an important cognitive factor that affects academic engagement. For example, Salanova et al. (2011) indicated that self-efficacy beliefs would affect student performance, participation in activities, and academic engagement. Studies have also found a significant relationship between self-efficacy beliefs and academic engagement (Zhen et al., 2017; Maricuţoiu and Sulea, 2019). Recently, it was also reported that academic self-efficacy partially mediated the effect of positive academic emotions on academic engagement (Lin et al., 2020). This is because students with high self-efficacy will do their best to achieve their goals and seek success by overcoming difficulties (Oriol-Granado et al., 2017). Thus, the following hypotheses are proposed:

Hypothesis 2: Academic passion is positively correlated with academic self-efficacy.

Hypothesis 3: Academic self-efficacy mediates the relationship between academic passion and academic engagement.

### Moderating Role of Teacher Developmental Feedback

The main goal of teacher feedback is to narrow the gap between students' understanding and performance (Hattie, 2009). Developmental feedback is informational feedback that helps recipients learn, develop, and improve (Zhou, 2003). Each student must be able to comprehend the meaning of teacher developmental feedback to self-assess what has been achieved and what needs improvement. The broaden-and-build theory posits that positive emotions may strongly inspire individuals' willingness to receive information (Fredrickson and Branigan, 2005). Therefore, we believe that the process by which academic passion affects academic engagement may be affected by teacher feedback. Management studies have illustrated that timely recognition and praise given to subordinates through superiors' developmental feedback inspired subordinates' work engagement (Su and Lin, 2018). In contrast to the developmental feedback of superiors, teacher developmental feedback is a way of communication that carries an emotional connection between teachers and students. In the field of education, several studies have shown that assessment feedback has a significant impact on college students' learning motivation and academic performance (Sendziuk, 2010; Yang and Lu, 2015). In addition, there is also a significant correlation between the nature of teacher feedback and student involvement in school activities (Pollock, 2011). Research has also found that different sources of formative assessment have great potential in facilitating student involvement in tasks (Zhang and Hyland, 2018). Thus, we propose the following hypothesis:

Hypothesis 4: Teacher developmental feedback positively moderates the relationship between academic passion and academic engagement. The higher the level of teacher developmental feedback, the stronger the positive relationship between academic passion and academic engagement.

**Figure 1:**
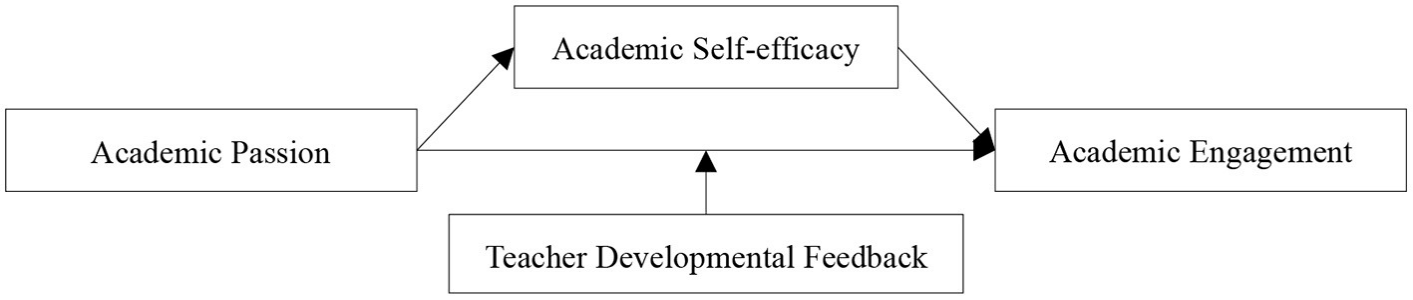
Research model.

Overall, by relying on the broaden-and-build and situated cognition theories, this study investigated the relationship between academic passion and academic engagement. The present findings will enable an improved understanding of the internal mechanism and boundary condition of the above relationship and provide theoretical guidance for improving the quality of students' academic engagement. Figure 1 shows the research model used in this study.

## Materials and Methods

### Participants and Procedure

In this study, based on the convenience sampling method, three colleges (a provincial key university and two ordinary undergraduate universities) in Henan Province, China, were selected for investigation, and the research participants included undergraduates. Self-report questionnaires were completed by participants after they were assured that their responses would remain anonymous and that their participation was voluntary. The study was approved by the Ethics Committee of the Henan Normal University.

To ensure the quality of data and reduce the impact of common method bias, we collected data at two time points. The survey data were collected in the form of two separate questionnaires with the help of related teachers. The survey lasted nearly a month and was conducted in two stages with an interval of 3 weeks. In the first stage, academic passion, academic self-efficacy, and demographic variables were measured. A total of 1,200 questionnaires were distributed during the first stage. There were 57 students who refused to participate in this study (4.75%). In the second stage, the participants who completed the questionnaire in the first stage were selected. In this stage, teacher developmental feedback and academic engagement data were collected. The questionnaire used a unique code matching method (required participants to provide the last six digits of their ID number) to match the two sets of questionnaires. After eliminating invalid questionnaires that were not answered carefully, 1,029 sets of valid questionnaires were successfully matched. The demographic survey results showed that the participants comprised 431 men (41.9%) and 598 women (58.1%). Their ages ranged from 18 to 24 years. There were 629 rural students (61.1%) and 400 urban students (38.9%).

### Measures

#### Academic Passion

We measured academic passion using the scale developed by Vallerand and Houlfort (2003). The Chinese version of the scale has good reliability when used to measure the academic passion of Chinese college students (e.g., Lin et al., 2019). The scale had a total of 19 items, including three subscales: passion criteria, harmonious passion, and obsessive passion. The passion criteria subscale used to determine whether an individual is passionate about a particular activity was selected in this study. This subscale had five items (e.g., “This activity is important for me”) that were rated on a 5-point Likert scale (1 = strongly disagree, 5 = strongly agree). Higher average scores indicate higher academic passion. Cronbach's alpha for the scale was 0.75.

#### Academic Self-Efficacy

We measured academic self-efficacy using a scale validated by Chen et al. (2001). The scale showed high reliability and was widely used to measure academic self-efficacy in Chinese studies (e.g., Cui et al., 2020). The scale included eight items (e.g., “I will be able to successfully overcome many challenges”) rated on a 5-point Likert scale. For each item, college students were required to determine the extent to which these descriptions fit their actual situation. The total score was the average value of the eight items. Higher scores indicate higher academic self-efficacy. Cronbach's alpha for the scale was 0.87.

#### Teacher Developmental Feedback

We measured teacher developmental feedback using a developmental feedback scale developed by Zhou (2003). The Chinese version of this scale has been validated to measure developmental feedback among Chinese participants (Geng et al., 2020). The scale included three items (e.g., “The teachers gave me feedback mainly to help me learn and improve”), and items were rated on a 5-point Likert scale. The total score was the average value of all the items. The larger the value, the greater the amount of developmental feedback given. Cronbach's alpha for the scale was 0.71.

#### Academic Engagement

We measured academic engagement using the shortened version scale developed by Schaufeli et al. (2006). The Chinese version of this scale has shown high reliability and has been widely used to measure academic engagement among Chinese college students (e.g., Zhao et al., 2021). The scale included nine items (e.g., “I am immersed in my study”) that were rated on a 5-point Likert scale. The higher the average score, the higher the level of academic engagement. The Cronbach's alpha for scale reliability was 0.89.

In addition, as gender and birthplace have a significant impact on college students' academic engagement (Yang and Zhang, 2016; Zhao et al., 2021), this study included these as control variables to minimize the influence of demographic characteristics. In this study, gender included men and women, and birthplaces included rural and urban areas.

### Data Analysis

SPSS 23.0 was used to test for common method bias and to conduct descriptive statistics, reliability analysis, and correlation analysis of the variables. Hypotheses were tested using the PROCESS macro of SPSS (Hayes, 2013, Model 5, 5,000 bootstrap resamples). In addition, Amos 23.0 was also used to test the common-method bias.

## Results

### Common Method Bias Test

The Harman single-factor test was used to statistically verify the presence of common method bias. The results reported that four factors had eigenvalues >1, and the first factor explained 34.01% of the total variance. The results did not exceed a critical value of 40%. In addition, the CFA results of the method-factor approach showed that the model fit of the four-factor model (χ^2^*/df* = 3.920, *CFI* = 0.928, *IFI* = 0.928, *TLI* = 0.918, *NFI* = 0.906, *RMSEA* = 0.053) did not significantly improve after adding the common method factors (χ^2^*/df* = 3.846, *CFI* = 0.930, *IFI* = 0.930, *TLI* = 0.920, *NFI* = 0.908, *RMSEA* = 0.053). The above method shows that there is no serious deviation in the general method.

### Correlations Between Primary Variables

Spearman correlations presented in Table 1 show that there was a significant positive relationship between academic passion and academic self-efficacy and teacher developmental feedback and academic engagement. These findings met the prerequisites for conducting a hypothetical test.

**Table 1:** Means, standard deviations, correlations, and reliabilities (in brackets).

		***M***	***SD***	**1**	**2**	**3**	**4**	**5**	**6**
1	Gender^a^	0.42	0.49						
2	Birthplace^b^	0.61	0.49	−0.05					
3	AP	3.22	0.65	−0.03	0.00	(0.75)			
4	AS	3.30	0.58	0.08^*^	−0.12^**^	0.41^**^	(0.87)		
5	TDF	3.55	0.61	−0.01	−0.01	0.25^**^	0.33^**^	(0.71)	
6	AE	2.92	0.65	−0.00	−0.03	0.62^**^	0.51^**^	0.16^**^	(0.89)

*AP, academic passion; AS, academic self-efficacy; TDF, teacher developmental feedback; AE, academic engagement. The numbers in brackets on the diagonal are Cronbach's alphas.*

* *p < 0.05,*

** *p < 0.01, N = 1,029 for College Students.*

a *Gender (“0” women; “1” men).*

b *Birthplace (“0” urban; “1” rural)*.

### Hypothesis Testing

We conducted a model with gender and birthplace as the control variables, academic passion as the independent variable, academic self-efficacy as the mediator, and teacher developmental feedback as the moderator to investigate the effect on academic engagement.

First, the moderating effect of teacher developmental feedback on the relationship between academic passion and academic engagement was examined. Table 2 (Model 1) shows that academic passion had a significant and positive effect on academic engagement (β = 0.61, *p* < 0.001), thus supporting Hypothesis 1. In addition, the interaction between academic passion and teacher developmental feedback was positively related to academic engagement (β = 0.16, *p* < 0.001). Therefore, teacher developmental feedback had a moderating effect on the relationship between academic passion and academic engagement.

**Table 2:** Results for the models.

**Variables**	**Model 1**			**Model 2**			**Model 3**		
	**AE**			**AS**			**AE**		
	**β**	***SE***	***t***	**β**	***SE***	***t***	**β**	***SE***	***t***
Constant	2.93	0.03	100.32^***^	3.34	0.03	110.79^***^	1.75	0.10	17.24^***^
Gender^a^	0.01	0.03	0.31	0.10	0.03	3.00^**^	−0.02	0.03	−0.77
Birthplace^b^	−0.04	0.03	−1.31	−0.13	0.03	−3.99^***^	0.00	0.03	0.12
AP	0.61	0.03	24.29^***^	0.37	0.03	14.76^***^	0.50	0.03	19.62^***^
TDF	0.01	0.03	0.22				−0.07	0.03	−2.80^**^
AP*TDF	0.16	0.04	4.56^***^				0.12	0.03	3.67^***^
AS							0.35	0.03	12.05^***^
*R^2^*	0.39			0.19			0.47		
*F*	131.17^***^			80.62^***^			149.00^***^		

*AP, academic passion; AS, academic self-efficacy; TDF, teacher developmental feedback; AE, academic engagement.*

** *p < 0.01,*

*** *p < 0.001, N = 1,029 for College Students.*

a *Gender (“0” women; “1” men).*

b *Birthplace (“0” urban; “1” rural)*.

**Figure 2:**
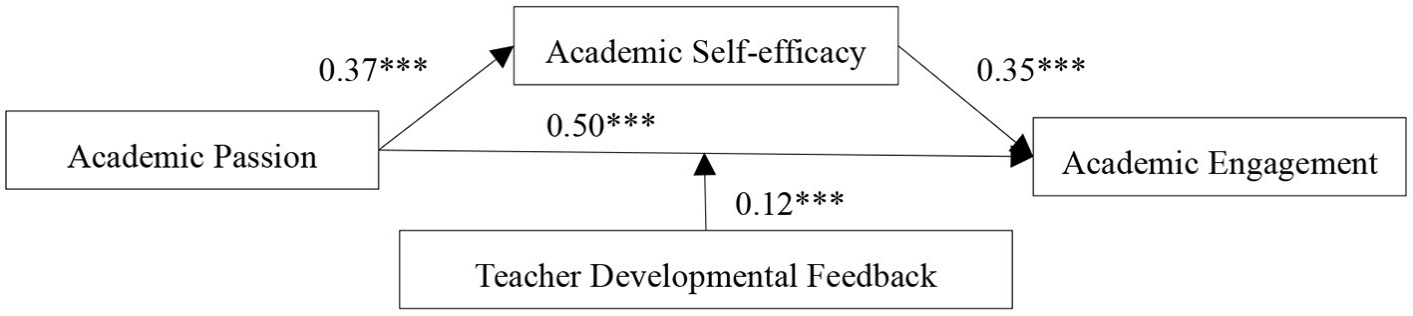
Roadmap of the influence of academic passion on academic engagement. ***p<0.001.

Second, academic self-efficacy was included in the model. Table 2 (Model 2) shows that academic passion had a significant impact on academic self-efficacy (β = 0.37, *p* < 0.001). Thus, Hypothesis 2 was supported. Table 2 (Model 3) and Figure 2 (below) show that academic passion had a positive influence on academic engagement (β = 0.50, *p* < 0.001), and that academic self-efficacy had a significant effect on academic engagement (β = 0.35, *p* < 0.001), thus supporting Hypothesis 3. In addition, it was found that academic self-efficacy partially mediated the relationship between academic passion and academic engagement. The interaction effect between academic passion and teacher developmental feedback on academic engagement was also significant (β = 0.12, *p* < 0.001). Therefore, Hypothesis 4 was also supported. Overall, the results showed that teacher developmental feedback positively moderated the relationship between academic passion and academic engagement.

Following prior research, we further observed the direct effect of academic passion on academic engagement at two standard deviations above and below the mean of the self-construal score. Consistent with our proposition, Table 3 shows that at one standard deviation below the mean, academic passion had a significant positive direct effect on academic engagement [95% confidence interval (CI), 0.36–0.49]. At one standard deviation above the mean of teacher developmental feedback, academic passion had a more significant positive direct effect on academic engagement (95% CI, 0.51–0.64). Compared to the indirect effect, academic passion mainly affected academic engagement through direct effects.

**Table 3:** Effects and 95% confidence intervals for model 3.

**Teacher developmental feedback**		**Academic passion on academic engagement**			
		**Effect**	**Boot SE**	**Boot LLCI**	**Boot ULCI**
Indirect effect	—	0.13	0.02	0.10	0.17
	−0.61	0.42	0.03	0.36	0.49
Direct effects	0.00	0.50	0.03	0.45	0.55
	0.61	0.57	0.03	0.51	0.64

*The path of indirect effect is “academic passion → academic self-efficacy → academic engagement,” and the path of direct effects is “academic passion → academic engagement.”*

**Figure 3:**
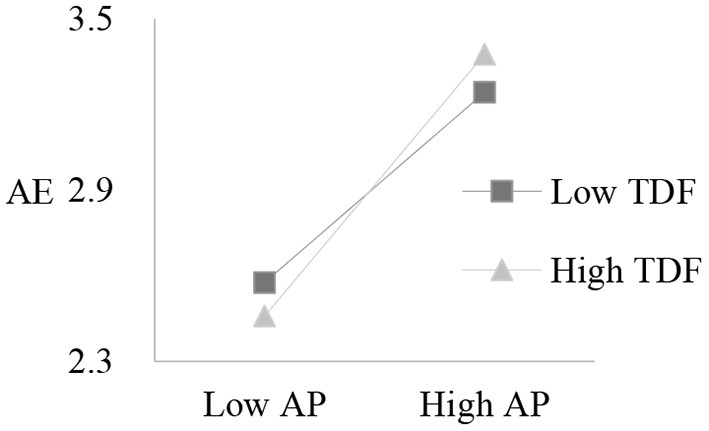
Interaction of academic passion and teacher developmental feedback on academic engagement. Low AP, low academic passion; High AP, high academic passion; Low TDF, low teacher developmental feedback; High TDF, high teacher developmental feedback; AE, academic engagement.

In addition, the simple slope analysis (Figure 3) confirmed that academic passion had a stronger effect on academic engagement under high teacher developmental feedback (β = 0.46, *t* = 21.43, *p* < 0.001) than under low teacher developmental feedback (β = 0.33, *t* = 15.65, *p* < 0.001), thus supporting Hypothesis 4. This showed that when students received more developmental feedback from teachers, their academic passion had a stronger positive predictive effect on academic engagement.

## Discussion

What remains to be answered is how academic passion contributes to academic engagement. Therefore, in this study, we attempted to reveal the underlying mechanism of the relationship between academic passion and academic engagement. The results showed that (a) academic passion had a positive effect on academic engagement, (b) academic self-efficacy partially mediated the relationship between academic passion and academic engagement, and (c) teacher developmental feedback moderated the relationship between academic passion and academic engagement. Thus, these findings suggested that the higher the level of developmental feedback, the stronger the positive relationship between academic passion and academic engagement.

First, this study tested whether academic passion could effectively predict academic engagement. Thus, it not only effectively promoted the development of the broaden-and-build theory in the field of education, but also expanded the scope of research on the factors affecting academic engagement. This study revealed the positive effects of academic passion from the perspective of positive psychology, and answered the theoretical questions “Is there a positive relationship between academic passion and academic engagement?” and “How does academic passion affect academic engagement?” This result is consistent with an earlier study that suggested a positive relationship between academic passion and engagement (Enwereuzor et al., 2016). Therefore, these findings suggest that students with academic passion can realize the value and significance of learning (Lin et al., 2019), thus they will invest more positive emotions, efforts, and concentration into learning. This suggests that students will have a higher level of academic engagement if they have a strong academic passion for learning. The stimulation of academic passion mainly depends on the students' love for learning itself, or the students' recognition of the external effects that learning can obtain (Vallerand et al., 2007). Therefore, teachers should be aware that learning itself and external motivation are key factors in fostering academic passion (Stoeber et al., 2011). Consequently, teachers can create a series of designs based on the following principles: the curriculum design needs to be rich and interesting, learning tasks must be challenging and innovative, the learning atmosphere must be autonomous, and the learning results must be rewarded. By incorporating these, teachers will be able to enhance students' academic passion and academic engagement.

Second, this study found the mediating role of academic self-efficacy and revealed the internal mechanism by which academic passion affects academic engagement. This is not surprising as prior research has shown that academic self-efficacy not only helps individuals undertake and make the necessary efforts to successfully complete various tasks, but also provides them with more personal resources to achieve good academic performance (Whannell et al., 2012). Thus, students with a higher sense of academic self-efficacy are motivated to use more learning strategies, improve their cognitive competency, and remain persistent when encountering learning challenges (Wright et al., 2012). Self-efficacious students therefore hold stronger beliefs in the successful completion of their learning goals, more investment, longer persistence, and an improved ability to cope with failure than students with low academic self-efficacy (Oriol-Granado et al., 2017). This finding thus suggests that a strong academic passion can enhance students' sense of academic self-efficacy in learning, enabling them to set positive goals, mobilize more resources, and invest more effort in learning, thus promoting the continuous improvement of academic engagement. According to Bandura, self-efficacy beliefs have stable (trait-like) and contextual components, which are dependent on variables such as recent performance, alternative experiences, interpersonal persuasiveness, or current physiological state (Bandura, 1997). Therefore, on the one hand, teachers should actively create a free, open, and error-free learning atmosphere; on the other hand, when students successfully overcome difficulties or obtain major achievements through independent efforts, teachers should give timely encouragement to improve students' academic self-efficacy.

Third, this study integrated teacher developmental feedback into the theoretical framework and identified the boundary condition in the relationship between academic passion and academic engagement. It was found that when teachers have more developmental feedback, the positive effect of students' academic passion on academic engagement was stronger. This can be explained as follows: teacher developmental feedback provides students with helpful information, assesses students' learning status, narrows the gap between students' understanding and performance (Hattie, 2009), satisfies the desire of students with high academic passion to obtain informational feedback, and ultimately improves their ability to participate in learning and academic engagement (Yang and Lu, 2015). In short, teachers' developmental feedback has a huge impetus in enhancing academically passionate students' academic engagement. The above research conclusions responded to researchers' appeal to reveal the positive effects of teacher developmental feedback and provided new ideas for further research on academic engagement in the future. The results of the present study highlighted that students with strong academic passion and academic self-efficacy also needed teacher developmental feedback to obtain higher academic engagement. Therefore, teachers should provide frequent, high-quality feedback, and create a supportive environment for students to actively participate in learning (Wang and Zhang, 2020). On the one hand, teachers need to be familiar with and grasp the art of feedback, carefully observe the student's learning situation, and add objective persuasion. On the other hand, in the education process, teachers need to establish good “online” and “offline” communication platforms to reduce the face cost of students and encourage them to speak freely about the problems they are facing in learning.

Finally, this study used Chinese college students as research subjects. Therefore, in the context of Chinese culture, this study investigated the relationship between academic passion and academic engagement. Previous studies have shown that students with different learning stages and cultural backgrounds have different levels of psychological and academic engagement (Martin et al., 2014; Yin, 2020). However, previous studies have mainly tested the impact of academic passion on academic engagement at the postgraduate stage (e.g., Lin et al., 2019) and in countries such as the United Kingdom (e.g., Stoeber et al., 2011), with few studies focusing on Chinese college students. Therefore, this study not only tested and enriched previous research finding, but also laid a foundation for future research on the influencing factors of academic engagement at different learning stages and in different cultures. The findings of the present study provide a theoretical reference for higher education in other countries.

## Limitations and Future Research

This study explored the mechanism of academic passion's impact on academic engagement, which provided several theoretical and practical guidance; however, certain limitations were also present. First, this study only selected three universities in Henan Province, China, for investigation. Constrained by the convenience sampling method, the representativeness of the sample declined. The scope of this investigation should be further expanded. Furthermore, this study focused on the impact of college students' academic passion on academic engagement. In the future, the academic engagement of primary and secondary school students can be further studied and new research findings are expected. Second, academic engagement data were collected only by students' self-reports; thus, response bias could have influenced the results. In the future, data such as teacher evaluations should be included for more comprehensive measurements. Third, this study revealed the internal mechanism from the perspective of cognitive processes; however, the mediating paths of motivation and other factors should also be investigated in the future. In terms of boundary conditions, future researchers should examine the potential strengthening or restrictive conditions of factors, such as the learning atmosphere, for undergraduates' academic engagement.

## Conclusion

The study emphasized the role of academic passion in enhancing college students' academic engagement. In addition, the findings showed that students with high academic passion had higher academic self-efficacy, which encouraged them to maintain a higher degree of academic engagement. Furthermore, the higher the level of developmental feedback, the stronger the positive relationship between academic passion and academic engagement. It is hoped that the findings will guide the development of interventions to increase students' academic passion, thus promoting increased academic engagement.

## Data Availability Statement

The raw data supporting the conclusions of this article will be made available by the authors, without undue reservation.

## Author Contributions

HZ conceived the research idea, structured and drafted the manuscript. XL and CQ collected and analyzed the data. All authors contributed to the article and approved the submitted version.

## Conflict of Interest

The authors declare that the research was conducted in the absence of any commercial or financial relationships that could be construed as a potential conflict of interest.
